# Validation of a Quantitative Ultrasound Texture Analysis Model for Early Prediction of Neoadjuvant Chemotherapy Response in Breast Cancer: A Prospective Serial Imaging Study

**DOI:** 10.3390/cancers17152594

**Published:** 2025-08-07

**Authors:** Daniel Moore-Palhares, Lakshmanan Sannachi, Adrian Wai Chan, Archya Dasgupta, Daniel DiCenzo, Sonal Gandhi, Rossanna Pezo, Andrea Eisen, Ellen Warner, Frances Wright, Nicole Look Hong, Ali Sadeghi-Naini, Mia Skarpathiotakis, Belinda Curpen, Carrie Betel, Michael C. Kolios, Maureen Trudeau, Gregory J. Czarnota

**Affiliations:** 1 Department of Radiation Oncology, Sunnybrook Health Sciences Centre, Toronto, ON M4N 3M5, Canada; 2 Department of Radiation Oncology, University of Toronto, Toronto, ON M5S 1A1, Canada; 3 Physical Sciences, Sunnybrook Research Institute, Toronto, ON M4N 3M5, Canada; 4 Division of Medical Oncology, Department of Medicine, Sunnybrook Health Sciences Centre, Toronto, ON M4N 3M5, Canada; 5 Department of Medicine, University of Toronto, Toronto, ON M5S 1A1, Canada; 6 Department of Surgical Oncology, Department of Surgery, Sunnybrook Health Sciences Centre, Toronto, ON M4N 3M5, Canada; 7 Department of Surgery, University of Toronto, Toronto, ON M5S 1A1, Canada; 8 Department of Medical Biophysics, University of Toronto, Toronto, ON M5S 1A1, Canada; 9 Department of Electrical Engineering and Computer Sciences, Lassonde School of Engineering, York University, Toronto, ON M3J 1P3, Canada; 10 Department of Medical Imaging, Sunnybrook Health Sciences Centre, Toronto, ON M4N 3M5, Canada; 11 Department of Medical Imaging, University of Toronto, Toronto, ON M5S 1A1, Canada; 12 Department of Physics, Toronto Metropolitan University, Toronto, ON M5B 2K3, Canada

**Keywords:** breast cancer, quantitative ultrasound, radiomics, machine learning, neoadjuvant chemotherapy, precision oncology

## Abstract

Background/Objectives: Patients with breast cancer who do not achieve a complete response to neoadjuvant chemotherapy (NAC) may benefit from intensified adjuvant systemic therapy. However, such treatment escalation is typically delayed until after tumour resection, which occurs several months into the treatment course. Quantitative ultrasound (QUS) can detect early microstructural changes in tumours and may enable timely identification of non-responders during NAC, allowing for earlier treatment intensification. In our previous prospective observational study, 100 breast cancer patients underwent QUS imaging before and four times during NAC. Machine learning algorithms based on QUS texture features acquired in the first week of treatment were developed and achieved 78% accuracy in predicting treatment response. In the current study, we aimed to validate these algorithms in an independent prospective cohort to assess reproducibility and confirm their clinical utility. Methods: We included breast cancer patients eligible for NAC per standard of care, with tumours larger than 1.5 cm. QUS imaging was acquired at baseline and during the first week of treatment. Tumour response was defined as a ≥30% reduction in target lesion size on the resection specimen compared to baseline imaging. Results: A total of 51 patients treated between 2018 and 2021 were included (median age 49 years; median tumour size 3.6 cm). Most were estrogen receptor–positive (65%) or HER2-positive (33%), and the majority received dose-dense AC-T (n = 34, 67%) or FEC-D (n = 15, 29%) chemotherapy, with or without trastuzumab. The support vector machine algorithm achieved an area under the curve of 0.71, with 86% accuracy, 91% specificity, 50% sensitivity, 93% negative predictive value, and 43% positive predictive value for predicting treatment response. Misclassifications were primarily associated with poorly defined tumours and difficulties in accurately identifying the region of interest. Conclusions: Our findings validate QUS-based machine learning models for early prediction of chemotherapy response and support their potential as non-invasive tools for treatment personalization and clinical trial development focused on early treatment intensification.

## Simple Summary

Some breast cancer patients do not respond to neoadjuvant chemotherapy, but it often takes several months (until the tumour is surgically removed) to identify who is not responding. This delay can prevent doctors from switching to a more aggressive treatment that could improve outcomes. Our research explores a non-invasive ultrasound technique that can detect early changes in the tumour within the first week of treatment. By using artificial intelligence to analyze these ultrasound images, we can predict which patients are unlikely to respond to chemotherapy much earlier in their treatment. This could help doctors personalize care sooner and improve outcomes for patients with breast cancer.

## 1. Introduction

Chemotherapy is an essential part of management of non-metastatic breast cancer and is typically recommended for patients with locally advanced disease (e.g., primary tumours ≥5 cm or lymph node involvement) or those at increased risk of distant failure (e.g., cT2 N0 HER2-positive or triple-negative disease) [1,2,3]. When indicated, chemotherapy is preferably administered before tumour resection, as neoadjuvant delivery enables tumour downstaging, improves operability, and facilitates surgical de-escalation [1,2,3]. In addition, neoadjuvant chemotherapy allows for in vivo assessment of treatment response and risk-adapted adjuvant strategies, particularly in patients with triple-negative or HER2-positive disease [4,5]. This is because patients with a poor response to neoadjuvant therapy are at higher risk of relapse and may benefit from intensified adjuvant treatment, whereas those who achieve a complete response have a lower recurrence risk and may avoid unnecessary treatment escalation [4,5,6].

Despite the advantages of neoadjuvant chemotherapy, the decision to modify systemic treatment is generally made only after tumour resection, once the full course of chemotherapy has been completed. This approach not only delays treatment intensification in non-responders but also limits the exploration of de-intensification strategies, such as reducing the number of chemotherapy cycles in early responders. Therefore, there is a pressing need to investigate imaging modalities capable of assessing treatment response early during neoadjuvant therapy, enabling real-time adaptation based on individual patient response.

Prospective studies have investigated the use of mid-treatment imaging modalities, such as ultrasound, magnetic resonance imaging (MR), and positron emission tomography–computed tomography (PET-CT), to support personalized treatment strategies [7,8,9,10,11,12]. For example, in the GeparTrio trial, patients who failed to achieve a >50% reduction in tumour size by ultrasound after two cycles of TAC neoadjuvant chemotherapy (docetaxel, doxorubicin, cyclophosphamide) were randomized to either continue TAC or switch to NX (vinorelbine and capecitabine) [8]. Although the adaptive strategy did not improve pathological complete response rates compared with the standard regimen, one potential explanation is the uncertainty surrounding the optimal imaging modality and timing for early response assessment. Therefore, this early adaptive approach remains promising and warrants further investigation.

Standard B-mode ultrasound, while attractive for its low cost and widespread availability, primarily detects anatomical changes (i.e., tumour shrinkage) that tend to occur later in the treatment course. Consequently, there is growing interest in imaging techniques that can identify earlier biological changes and support timely treatment decisions. In this context, quantitative ultrasound (QUS) has emerged as a promising method for early response assessment [13,14]. QUS detects microstructural alterations in tissue—such as changes in cellular density, organization, and elasticity—through quantitative analysis of backscattered ultrasound signals [15,16,17,18,19,20,21,22]. These biologically changes precede visible reductions in tumour size and can be detected as early as the first week of treatment [17].

We previously conducted a prospective observational imaging study involving 100 patients with breast cancer receiving neoadjuvant chemotherapy (referred to as the “Development Cohort”) [15,16]. Patients underwent QUS imaging before treatment and at weeks 1, 4, and 8 of chemotherapy. The goal was to identify QUS parameters predictive of treatment response and to develop machine learning models to enable treatment personalization. Using texture analysis of the primary tumour, we created predictive algorithms that differentiated responders from non-responders with accuracies of 79% at week 1, 90% at week 4, and 92% at week 8 [15,16]. Although these models demonstrated high predictive performance, they were not validated in independent cohorts, which is a critical step before clinical implementation.

Building on these findings, a separate prospective clinical trial (ClinicalTrials.gov: NCT04050228) was launched to evaluate the feasibility of using QUS-radiomics models to guide chemotherapy personalization (referred to as the “Validation Cohort”). In this study, patients underwent QUS imaging at the same pre-specified intervals: baseline and at 1, 4, and 8 weeks after starting chemotherapy. In the present analysis, our aim is to validate the week 1 prediction model in this independent cohort, assess its reproducibility and predictive accuracy, and support its integration into future adaptive neoadjuvant trials.

## 2. Methods

### 2.1. Study Population

Both the development (7) and validation cohorts shared similar inclusion criteria, tumour response definition, and QUS methodology, which will be described as follows. Patients were included if they had biopsy-proven breast cancer and primary tumours measuring at least 1.5 cm in size, for whom neoadjuvant chemotherapy was deemed adequate per standard of care, and underwent QUS on the first week of neoadjuvant chemotherapy. This study received approval from the institutional ethics committee and was registered with ClinicalTrials.gov (NCT04050228) on 22 July 2019. All patients provided written consent before study participation.

### 2.2. Tumour Response Definition

MR was acquired at baseline (staging MR) before chemotherapy started in order to assess tumour extension and serve as a measurement reference for tumour response. Patients were stratified as responders (R) and non-responders (NR) using a modified response grading system based on the clinical/pathological tumour response determined at the end of their neoadjuvant treatment [15,16]. The response category encompassed a complete disappearance of disease or a reduction in the diameter of target lesions by at least 30%, or a reduction in cellularity to <5% in the tumour bed (in cases of invasive disease), regardless of size, or the complete disappearance of all target lesions. This definition includes both partial and complete responders. The non-response category included a decrease in tumour size of less than 30%, accompanied by no significant changes in tumour cellularity, encompassing both stable disease and progressive disease.

### 2.3. Quantitative Ultrasound and Texture Parameters Estimation

Radio frequency (RF) ultrasound data were acquired volumetrically from breast tumours using a Sonix RP clinical system (Ultrasonix Medical Corporation, Vancouver, BC, Canada), with a L14-5/60 linear transducer (central frequency 6.5 MHz, bandwidth range 3.0–8.5 MHz) before starting the treatment (week 0) and at week 1 during treatment. Several image planes were acquired from the primary tumour at 0.5 cm intervals with the transducer focus at the mid-depth of the tumour. A Fast Fourier Transform (FFT)-based algorithm was applied on RF data to construct quantitative ultrasound parametric images including the mid-band fit (MBF), average acoustic scatter concentration (AAC), acoustic scatter diameter (ASD), spectral slope (SS), spectral intercept (SI), spectral average slope (SAS) using a sliding window analysis. The reference phantom method was used to remove any ultrasound system dependencies in QUS parameters estimation. RF-Data associated with primary images were used to construct QUS parametric images for each of the QUS parameters above.

Subsequently, a grey-level co-occurrence matrix (GLCM) based texture analysis technique was applied on QUS parametric images. The GLCM represents the statistical angular relationship between neighbouring pixels, as well as the distance between them. Four texture features, including contrast (CON), correlation (COR), homogeneity (HOM), and energy (ENE), were determined based on the statistical information provided by GLCM analysis. QUS parametric maps of the MBF, SS, SI, SAS, ASD and AAC from tumour regions underwent a GLCM-based texture analysis process to extract these four texture features. A total of 31 features including 6 means of QUS parameters, 24 texture features and attenuation were determined from tumour data. The changes in those feature (ΔQUS_week1_ = QUS_week1_ − QUS_week0_) at week 1 were used to predict the breast cancer treatment response using QUS-radiomics based treatment response prediction model.

### 2.4. Classification Model Algorithm

The multi-parametric classification model proposed in our development cohort [15,16] was used to predict cancer treatment response early at week 1 after starting treatment. That treatment response prediction model was developed based on a 100-patient QUS and a texture feature data set (81 responders and 19 non-responders) acquired with the Ultrasonix-RP system, using a radial basis function support vector machine classifier (SVM-RBF) [15,16]. That treatment response prediction model distinguished responder and non-responders at week 1 after treatment initiation with accuracy of 78%, sensitivity of 77%, specificity of 80%, and area under the curve of 0.79 [15,16]. During treatment response prediction model development, a sequential feature selection method was used to select most relevant features from total 31 features for tumour response classification. The optimal features identified through this process were ΔSS-COR, ΔMBFF-ENE, and ΔSAS-HOM. This model was subsequently applied to the QUS and texture-based parameters estimated from ultrasound data of patients included in this validation cohort.

### 2.5. Statistical Analysis

Since the current study was for validating QUS-radiomics based breast cancer treatment response prediction model, a sample size calculation was based on convenience without formal statistical analysis but represents a test set 50% of the training developmental set. Descriptive analysis was performed to study the patient, disease, treatment-related factors and response rates. We computed the sensitivity, specificity, positive predictive value, and negative predictive value in the validation cohort. Sensitivity was determined as the percentage of individuals correctly identified as non-responders among those who were actually non-responders. Specificity represented the percentage of responders correctly identified as responders. The positive predictive value indicated the percentage of patients identified as non-responders after neoadjuvant chemotherapy among those predicted to be non-responders. The negative predictive value was calculated as the percentage of patients identified as responders among those predicted to be responders. Image preprocessing, feature extraction, and radiomics model implementation were carried out using MATLAB R2020a (MathWorks, Natick, MA, USA). Other statistical tests were performed using IBM SPSS version 22 (IBM Corporation, Armonk, NY, USA). Standard statistical methods were used to calculate test performance.

## 3. Results

The validation cohort consisted of 51 patients (Table 1). Patients were treated between June 2018 and July 2021. The median age for the cohort was 49 years (range, 27–80) and the median tumour size was 3.6 (1.7–12). A total of 43% of tumours (n = 22/51) were ER/PR+ HER2−, 22% (n = 11/51) were ER/PR+ HER2+, 12% (n = 6/51) were ER/PR− HER2+, and 24% (n = 12/51) triple-negative. Most patients underwent dose-dense AC-T (n = 34, 67%) or FEC-D (n = 15, 29%) chemotherapy ± trastuzumab.

### 3.1. Accuracy of Prediction Model

**Figure 1:**
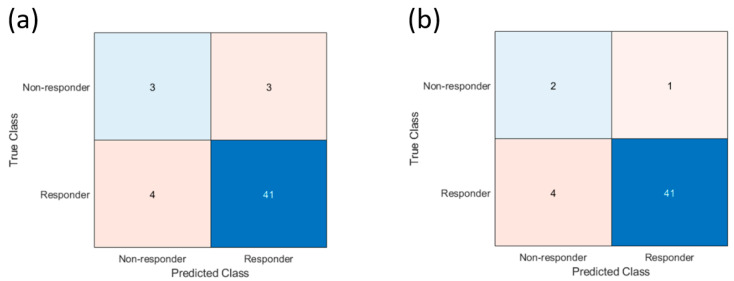
Confusion matrix of treatment response prediction results based on Week 1 quantitative ultrasound-radiomics model. (**a**) Analysis for the whole cohort (**b**) Exploratory analysis excluding 3 patients that did not complete the standard chemotherapy regiment.

Statistical analyzing using unpaired *t*-tests was performed in order to compare changes in mean QUS values (ΔQUS_week1_ = QUS_week1_ − QUS_week0_) and texture parameters between responder and non-responders. None of the changes in the estimated parameters demonstrated significant difference between two response groups. A support vector machine classifier algorithm predicted 45 patients would respond and 6 patients would not respond to chemotherapy. The algorithm correctly identified 41 out of the 45 responders and accurately classified 3 out of the 6 non-responders. This yielded an area under the curve (AUC) of 0.71, accuracy of 86%, specificity of 91%, sensitivity of 50%, negative predictive value of 93%, and positive predictive value of 43% (Figure 1, Table 2).

**Figure 2:**
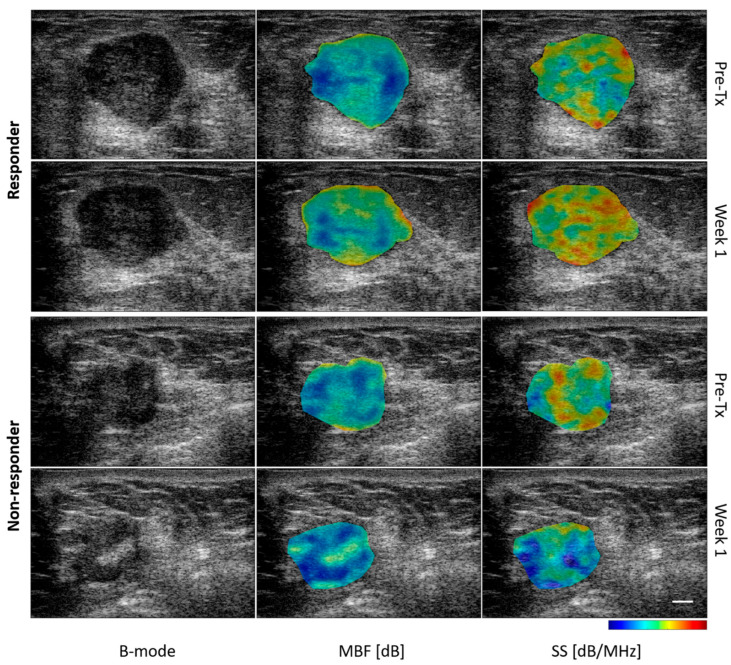
Representative B-mode, mid-band fit (MBF) and spectral slope (SS) parametric images at baseline (pre-Tx) and after the first week of treatment for one patient from responder and non-responder group. The scale bar in the ultrasound images represents 5 mm. The colour bar represents the scale for the MBF of −10 to 30 dB, and for SS parameter of −6 to 2 dB/MHz.

**Figure 3:**
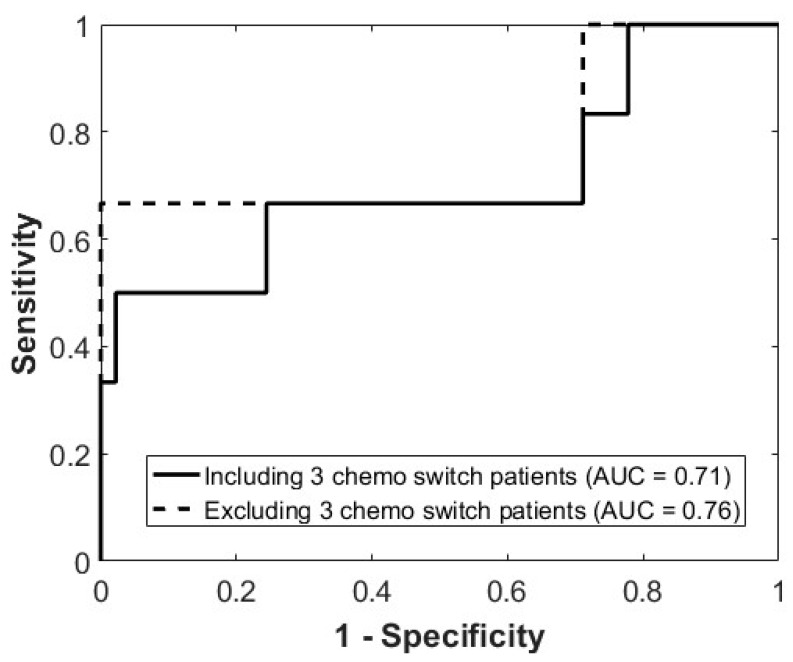
Receiver Operating Characteristic (ROC) curves illustrating model performance for predicting treatment response. The solid line represents the model including 3 chemotherapy switch patients (AUC = 0.71), while the dashed line excludes these 3 patients (AUC = 0.76).

We conducted an exploratory analysis by excluding the three patients who did not complete the full standard chemotherapy regimen, as incomplete standard chemotherapy treatment could lead to misclassification. In this analysis, the prediction algorithm exhibited an AUC of 0.76%, accuracy of 90%, specificity of 91%, sensitivity of 67%, negative predictive value of 98%, and positive predictive value of 33% (Table 2). Figure 2 exemplifies cases in which patients were accurately predicted to respond or non-respond to chemotherapy at week 1. Figure 3 presents the Receiver Operating Characteristic (ROC) curves, demonstrating the model’s performance in predicting treatment response.

### 3.2. Misclassified Patients

**Figure 4:**
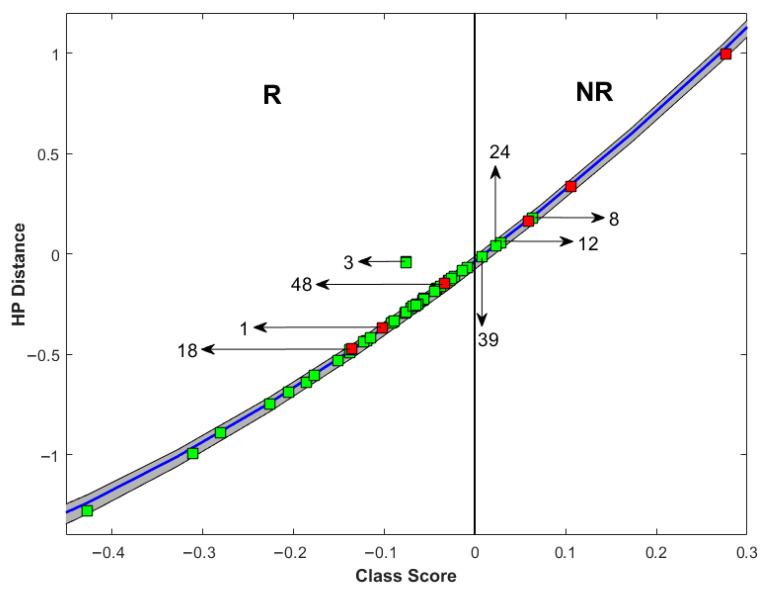
Treatment response prediction displayed using distance of new patient’s feature point from the Week 1 quantitative ultrasound-radiomics support vector machine model hyperplane and classification score (posterior probability). R indicates the zone (positive class score) for treatment responder prediction and NR indicates the zone (negative class score) for non-responder prediction. Green squares represent the true responder patients and red squares the true non-responder patients assessed on surgical pathology. Misclassified patient IDs are included and identified by arrows.

Figure 4 provides a comprehensive overview of patients who were predicted to either respond or not respond to chemotherapy, along with their actual final response classification on an individual patient basis. Patients with a negative class score were predicted to be responders, while those with a positive score were predicted to be non-responders. Actual responders are represented in green, while non-responders are in red. The figure illustrates eight patients whose response to chemotherapy was misclassified. Notably, these patients tended to have class scores closer to zero (border of responder/non-responder classification), indicating a potential indeterminate zone in the model’s classification capability in these cases or representing cases in which tumour definition was challenging. Upon individually reviewing the misclassified cases, it was observed that they often had heterogeneous, distorted, and poorly defined tumours. This characteristic made it difficult to select the region of interest, ultimately affecting accuracy. Moreover, as the scans were acquired in the first week of chemotherapy initiation, for some patients, this timing was not sufficient to detect microstructural changes that could inform response, resulting in suboptimal performance.

## 4. Discussion

Neoadjuvant chemotherapy provides an opportunity to assess tumour response in vivo, allowing clinicians to evaluate treatment effectiveness before surgery and potentially adapt therapy based on response. Researchers have focused on identifying imaging biomarkers that can predict response to neoadjuvant chemotherapy using pretreatment imaging modalities [23,24,25,26,27,28,29]. For instance, Xu et al. [24] developed a deep learning model using pretreatment MRI to predict pathologic complete response (pCR) to neoadjuvant therapy, achieving an area under the curve (AUC) of 0.76. While these findings are encouraging, relying solely on pretreatment imaging does not account for dynamic biological changes that occur during therapy [30]. Therefore, evaluating radiomic features after the initiation of chemotherapy may yield more accurate predictions of treatment response. In this context, our research has focused on extracting and analyzing radiomic features from mid-treatment quantitative ultrasound (QUS) imaging. This approach is supported by preclinical evidence showing that neoadjuvant chemotherapy can induce tumour cell death as early as 24 h after treatment initiation. These early changes include cellular fragmentation, aggregation, and chromatin condensation [20,21,22], processes that alter the tumour’s microstructure in ways that QUS can detect non-invasively [15,16,17,18,19].

Leveraging machine learning algorithms, we previously used support vector machine classifier algorithms that utilize changes in QUS to predict responses to neoadjuvant chemotherapy and identified accuracies ranging from 77% to 79% [16]. However, the application of machine learning algorithms involves a two-stage process: first, creating a classification algorithm using training data, and second, testing the model with a distinct data set. Therefore, in the study here, we performed internal validation of our radiomic model using a prospective and entirely independent cohort. Our results demonstrated strong predictive performance, achieving an overall accuracy of 86–90% and an AUC of 0.71–0.76.

Developing a non-invasive tool to predict chemotherapy response early in the disease course holds great significance for advancing personalized medicine. It has practical applications in clinical settings, allowing for individualized chemotherapy regimens. For instance, for patients predicted not to respond to chemotherapy, medical oncologists can use this information to consider treatment intensification, potentially converting non-responders into responders and improving operability. For example, an ongoing adaptive chemotherapy phase II clinical trial (Clinicaltrials.gov: NCT04050228) is investigating the feasibility of this approach, with future steps potentially including randomized clinical trials to assess the benefits of treatment intensification for patients predicted not to respond to chemotherapy. On the other hand, early response assessment can also be utilized to de-intensify treatment in responders, aiming to minimize toxicity while preserving oncologic outcomes. For example, in the PHERGain phase 2 randomized clinical trial, patients with HER2-positive breast cancer who demonstrated a metabolic response on mid-treatment PET scans were able to safely omit chemotherapy and receive exclusive dual HER2 blockade, without compromising invasive disease-free survival [9]. Using a different strategy, the single-arm phase 2 TRAIN-3 study investigated early treatment de-escalation based on mid-treatment MRI findings. Among patients with HER2-positive, hormone receptor–negative breast cancer, approximately one-third achieved a complete pathological response after only three cycles of chemotherapy. In these early responders, neoadjuvant therapy was safely discontinued ahead of schedule, leading to reduced treatment-related toxicity without adversely affecting event-free survival [7].

Our study has several strengths including the development and validation of predictive models in prospective cohorts with a similar patient population undergoing similar chemotherapy regimens (Table 1) [16]. Some patients misclassified at 1 week after chemotherapy had high DCIS components or presented mucinous features, both of which can confound the QUS response monitoring algorithms. These algorithms were developed with classifiers at various intervals after the commencement of chemotherapy (in this case, 1 week) and are weighted to correspond with the initial phase of anthracycline–taxane-based chemotherapy regimens (e.g., during the AC phase of AC-paclitaxel and during the FEC phase of FEC-docetaxel). Consequently, late responses to the second phase may not be adequately accounted for, although responses to the two phases typically exhibit consistency. Nevertheless, our study does have limitations, including a small number of non-responding patients. This limitation constrains the statistical significance or our findings. The lower positive predictive value (PPV) is attributed to this limited number of non-responders, indicating a prevalence rate of 12% in our patient population. Similarly, in our previous study [31], we developed a treatment response prediction model based on ultrasound data collected at week 4 and mode was validated with limited patients including 48 responder and 7 non-responders. However, accuracy of treatment response prediction was 90% due to the more pronounced difference in the change in QUS and texture parameters between the two response groups at that time point. The primary objective of the current study is to validate the prediction model based on QUS and texture parameters estimated very early during treatment at week 1. While this early time point is clinically relevant, the small number of non-responders remains a significant limitation and highlights the need for further studies with larger and more balanced cohorts to confirm these findings and improve the robustness of the predictive model.

## 5. Conclusions

In conclusion, the work here validated in an independent internal cohort a machine learning-driven radiomic predictive model that was based on QUS in a prospective and entirely independent cohort. This validation reinforces the model’s robustness as a non-invasive tool for the early prediction of tumour response to neoadjuvant chemotherapy, potentially enabling personalized treatment strategies. External validation in larger cohorts is needed to confirm the applicability of our model in other populations.

## Figures and Tables

**Table 1:** Patient, disease, and treatment characteristics for patients in the development and validation cohorts.

Characteristic		Development Cohort (n = 100)	Independent Validation Cohort (n = 51)
Age (years)		49 (29–84)	49 (27–73)
Primary tumour size (cm)		5.3 (1.6–12.8)	3.6 (1.7–12)
Histological Type			
	IDC	95 (95%)	50 (98%)
	ILC	5 (5%)	1 (2%)
Estrogen Receptor status			
	Positive	63 (63%)	33 (65%)
	Negative	37 (37%)	18 (35%)
HER2 Status *			
	Positive	24 (24%)	17 (33%)
	Negative	73 (73%)	34 (67%)
Planned Chemotherapy Regimen			
	AC-T ± Trastuzumab	59 (59%)	34 (67%)
	FEC-D ± Trastuzumab	29 (29%)	15 (29%)
	Others (i.e., TC or TH)	12 (12%)	2 (4%)

Categorical variables were presented as counts and percentages of the entire population. Continuous variables were described as median with a range. Age was presented in years and tumour size in cm. * HER2 status was unknown for 3 patients.

**Table 2:** Classifier performance of the quantitative ultrasound-radiomics model at week 1 of neoadjuvant chemotherapy in the development and validation cohorts.

Parameter	Development Cohort (95% CI)	Validation Cohort (95% CI)	
		Entire Cohort	Exploratory Analysis
Sensitivity	77% (75–78%)	50% (50–50%)	67% (65–69%)
Specificity	80% (78–82%)	91% (87–94%)	91% (87–95%)
Positive predictive value	52% (51–52%)	43% (43–43%)	33% (33–33%)
Negative predictive value	93% (91–94%)	93% (89–96%)	98% (94–100%)
Accuracy	78% (76–79%)	86% (83–89%)	90% (86–93%)
AUC	0.79 (0.78–0.80)	0.71 (0.68–0.73)	0.76 (0.73–0.79)

Confidence intervals for sensitivity, specificity, and accuracy are “exact” Clopper–Pearson confidence intervals. Confidence intervals for predictive values are standard logit confidence intervals.

## Data Availability

Data are stored in an institutional repository and will be made available on request to the corresponding author following institutional ethics committee protocols.

## Abbreviations

Acoustic scatter diameter (ASD), energy (ENE), area under the curve (AUC), average acoustic scatter concentration (AAC), contrast (CON), correlation (COR), fast Fourier transform (FFT), grey-level co-occurrence matrix (GLCM), homogeneity (HOM), magnetic resonance imaging (MR), mid-band fit (MBF), non-responders (NR), quantitative ultrasound imaging (QUS), radial basis function support vector machine classifier (SVM-RBF), radio frequency (RF), responders (R), spectral average slope (SAS), spectral intercept (SI), spectral slope (SS).
